# Regulatory Effect of Electroacupuncture on Hypothalamic Serotonin and its Receptor in Rats with Cerebral Ischemia

**DOI:** 10.2174/1567202620666230612110156

**Published:** 2023-08-28

**Authors:** Tongjun Ma, Chenyu Li, Zeyin Nie, Huachun Miao, Feng Wu

**Affiliations:** 1 Department of Human Anatomy, Wannan Medical College, Wuhu, Anhui, 241002, China

**Keywords:** Ischemic brain injury, hypothalamus, EA, 5-HT, 5-HTT, 5-HT2A

## Abstract

**Background:**

Previous studies have shown that the neurological damage caused by middle cerebral artery occlusion (MCAO) is not only limited to local infarction but can also cause secondary damage in distant sites, such as the hypothalamus. 5-hydroxytryptamine (5-HT)/ 5-HT transporter (5-HTT) and 5-HT receptor 2A (5-HT2A) are important in the treatment of cerebrovascular diseases.

**Objective:**

This study aimed to study the effect of electroacupuncture (EA) on the expression of 5-HT, 5-HTT, and 5-HT2A in the hypothalamus of rats with ischemic brain injury and to explore the protective effect and potential mechanism of EA on the secondary injury of cerebral ischemia.

**Methods:**

Sprague-Dawley (SD) rats were randomly divided into three groups: sham group, model group, and EA group. The permanent middle cerebral artery occlusion (pMCAO) method was used to induce ischemic stroke in rats. In the EA group, the Baihui (GV20) and Zusanli (ST36) points were selected for treatment, which was administered once per day for two consecutive weeks. The neuroprotective effect of EA was evaluated by nerve defect function scores and Nissl staining. The content of 5-HT in hypothalamus was detected by enzyme linked immunosorbent assay (ELISA), and the expression of 5-HTT and 5-HT2A were detected by Western blot.

**Results:**

Compared with that in the sham group, the nerve defect function score in the model group rats was significantly increased, the hypothalamus tissue showed obvious nerve damage, the levels of 5-HT and the expression of 5-HTT were significantly reduced, and the expression of 5-HT2A was significantly increased. After 2 weeks of EA treatment, the nerve defect function scores of pMCAO rats were significantly reduced, the hypothalamic nerve injury was significantly reduced, the levels of 5-HT and the expression of 5-HTT were significantly increased, and the expression of 5-HT2A was significantly decreased.

**Conclusion:**

EA has a certain therapeutic effect on hypothalamic injury secondary to permanent cerebral ischemia, and its potential mechanism may be closely related to the upregulation of 5-HT and 5-HTT expression and the downregulation of 5-HT2A expression.

## 1 INTRODUCTION

Previous studies have shown that the nerve injury caused by middle cerebral artery occlusion (MCAO) is not only limited to local infarction but can also cause secondary injury to distant regions, such as the hypothalamus, thalamus, substantia nigra, cerebellum, and spinal cord [1, 2]. Studies have shown that transient middle cerebral artery occlusion (tMCAO) in rats can cause apoptosis and autophagy in cells in the hypothalamus. In addition, hypothalamic ischemia and inflammation may also lead to multiple organ dysfunction syndrome [3-5].

Some studies have shown that after tMCAO injury, the degree of fiber projection at the infarct site is reduced, reducing 5-hydroxytryptamine (5-HT) levels in the frontal cortex, hippocampus, and thalamic nucleus and changing the level and function of other neurotransmitters, leading to metabolic dysfunction [6]. 5-HT is a typical neurotransmitter in the central nervous system that can regulate neuronal plasticity, neuronal proliferation and differentiation, and synaptogenesis. The levels of 5-HT in cortical tissue around the infarcted area of permanent middle cerebral artery occlusion (pMCAO) rats are significantly reduced [7]. Other studies have shown that pMCAO causes more severe brain tissue damage than tMCAO [8]. In ischemic cerebrovascular diseases, the reuptake and metabolism of 5-HT and changes in the 5-HT transporter (5-HTT) in the brain may directly change the activity of 5-HT neurons in the brain [9]. 5-HTT is the transporter with the highest affinity for 5-HT, which widely exists in the limbic system. Polygonum multiflorum Thunb extract can inhibit the abnormal increase in 5-HT receptor 2A (5-HT2A) levels in the hippocampus of tMCAO rats and gradually restore their cognitive function [7]. Xiaoyao-jieyu-san can upregulate the levels of 5-HT in the midbrain limbic system and downregulate the level of 5-HT2A, which can improve post-stroke depression [10]. Therefore, 5-HT/5-HT2A and 5-HTT are of great significance in the treatment of cerebrovascular diseases.

Numerous clinical and basic studies have shown that electroacupuncture (EA) at Baihui (GV20) and Zusanli (ST36) is effective in treating cerebral ischemia, but its mechanism is still unclear [11, 12]. At present, there has been no research report on the neuroprotective effect of EA on pMCAO secondary hypothalamic injury by regulating the 5-HT/5-HT2A signal pathway. Here, we hypothesized that EA plays a protective role in pMCAO secondary brain injury by regulating 5-HT/5-HT2A and 5-HTT.

This study investigated the role of 5-HT/HT2A in pMCAO secondary to hypothalamic injury and the potential mechanism in the EA treatment of ischemic brain injury.

## 2 MATERIALS AND METHODS

### 2.1 Animals

Forty-five Specific-pathogen-free (SPF) male Sprague-Dawley (SD) rats weighing 180~220 g were provided by the same supplier with license No. SCXK (Shanghai) 2018-0004. The rats were randomly divided into the sham group, model group, and EA group, with 15 rats in each group. During the experiments, the rats were kept in rooms with an appropriate temperature (24 ± 2°C) and natural light, and they were free to eat and drink water. The rats were treated in accordance with the regulations of the Experimental Animal Welfare and Ethics Committee of the Wannan Medical College on the Use of Animals and Ethics (No. LLSC-2021-025). We were committed to making every effort to minimize the suffering of the rats during the experiments.

### 2.2 Establishment of the pMCAO Model

The rat model of focal cerebral ischemia was induced by pMCAO. The rats were anesthetized with an intraperitoneal injection of anesthesia by sodium pentobarbital (30mg/kg) and were then placed on a clean surgical table. The right common carotid artery (CCA), external carotid artery (ECA), internal carotid artery (ICA), and vagus nerve were separated by fine glass needles through the anterior median cervical incision. The proximal ends of the ICA were ligated. A suture was inserted into the ICA until the head of the suture arrived at the origin of the middle cerebral artery (MCA). Finally, the incision was sutured.

### 2.3 EA Treatment

Rats in the EA group were stimulated 24 h after modeling. EA was performed on the GV20 and ST36 at a frequency of 2/10 Hz, 30 min once per day for 14 consecutive days.

### 2.4 Neurological Deficit Test

Based on Longa’s scoring method for neurologic functional deficits, the rats were scored after they naturally awoke from anesthetization, and symptoms of the neurologic functional deficits were scored as follows. 0 points: no nerve function defect; 1 point: the paw on the paralytic side cannot be fully extended; 2 points: when walking, the rat circles to the paralytic side; 3 points: when walking, the rat body topples to the paralytic side; 4 points: unable to walk spontaneously, loss of consciousness.

### 2.5 Nissl Staining

Samples from five rats in each group were cut into paraffin sections with a thickness of 5 µm and stained according to the instructions of the Nissl staining kit. Neutral gum film was used. After pictures were taken, the histological analysis of Nissl-positive cells was performed using ImageJ software (United States National Institutes of Health).

### 2.6 5-HT Enzyme-linked Immunosorbent Assay (ELISA) **Test**

The hypothalamus was collected from five rats in each group, and the level of 5-HT in the hypothalamus was determined according to the instructions of the ELISA kit (Shanghai Enzyme-Linked Biotechnology Co., Ltd, YJ603930, China).

### 2.7 Western Blotting Test

The right hypothalamus was taken from another five rats in each group. After tissue homogenization, lysis buffer was used to extract the total protein. Equal amounts of total protein (80µg) were loaded on 10% sodium dodecyl sulfate-polyacrylamide gel electrophoresis (SDS-PAGE) gels and separated for 1.5 h. The proteins were transferred to Polyvinylidene difluoride (PVDF) membranes at a constant current for 1.5 h. Blocking was performed for 1h at room temperature with 5% nonfat milk. The membranes were then incubated overnight at 4 after tissue homage (Wuhan Boster Biological Technology, PB0442, 1:1000, China) and anti-5-HT2A (Wuhan Boster Biological Technology, PB0599, 1:1000, China) antibodies. The membranes were then washed in Tris-Buffered Saline Tween-20 (TBST) and incubated in TBST containing horseradish peroxidase-conjugated anti-rabbit immunoglobulin G (IgG) at a 1/1,000 dilution for 1 h at room temperature. After three washes in TBST, the membrane was treated with enhanced chemiluminescence (ECL) Substrate (ThermoFisher) for chemiluminescent detection with an Amersham Imager 600 (Amersham). Finally, the relative gray values of the target proteins and internal reference protein were respectively measured by Image J Software.

### 2.8 Statistical Analysis

All data were statistically analyzed with SPSS 18.0 software. Graphical illustrations were made using GraphPad Prism 9.0 software. Measurement data presented as the mean were statistically analyzed with SPSS 18.0 software used for comparisons among multiple groups, and pairwise comparisons were conducted using the least significant difference (LSD) method. *p* <0.05 was considered statistically significant.

## 3 RESULTS

### 3.1 EA has a Neuroprotective Effect against Hypothalamic Injury Secondary to pMCAO

**Fig. (1):**
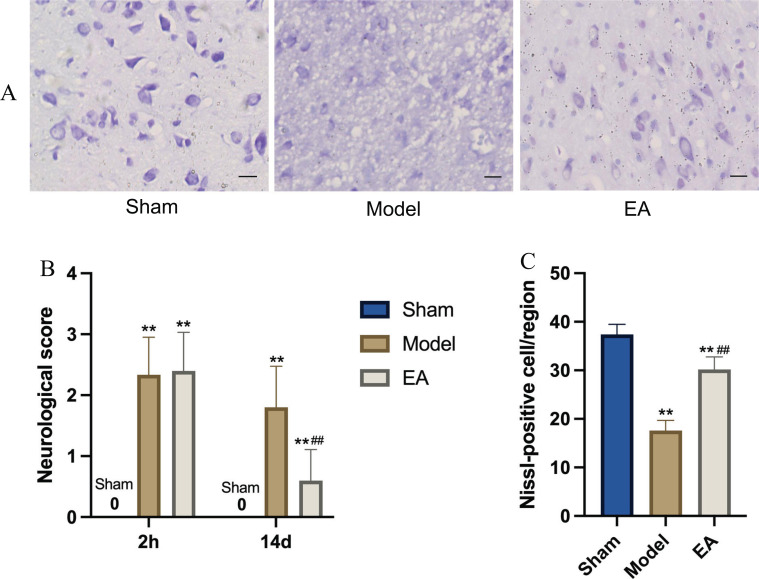
(**A**) EA has a neuroprotective effect against hypothalamic injury secondary to pMCAO. Representative images of Nissl Staining in the hypothalamus of rats in each group (y400, Scale bar=20 µm, n=15/group) showed that pMCAO led to neuronal damage, and EA treatment reversed these changes; (**B**) Comparison of neurological function scores of rats in each group. The Longa score was recorded to assess neurological deficits in rats. The results suggest that EA significantly alleviated neurological deficits in rats with cerebral ischemia; (**C**) Comparison of the number of Nissl positive neurons in rats of each group. Nissl Staining results showed that Nissl-positive cells were significantly reduced in the model group, while the Nissl-positive cells were increased in the EA group. *vs.* Sham group, **p <0.01; *vs.* Model group, ^##^*p* <0.01. The results are presented as the mean ± SEM. Student’s t-test.

After being subjected to pMCAO, the rats showed obvious hypothalamic injury. Compared with those in the sham group, the neurological deficit scores increased significantly in the model group (*p <*0.05), and the number of Nissl-positive cells was significantly reduced in the model group (*p <*0.05) (Figs. **1A**-**C**). This finding suggests that pMCAO can cause secondary hypothalamic injury. Hypothalamus injury in pMCAO rats was significantly reduced after EA treatment. Compared with that in the model group, the nerve defect functional score in the EA group was significantly decreased (*p <*0.05), and the number of Nissl-positive cells increased significantly (*p <*0.05) (Figs. **1A**-**C**). These results showed that EA treatment has a neuroprotective effect against hypothalamic injury secondary to pMCAO.

### 3.2 EA can Increase the Levels of 5-HT in the Hypothalamus after pMCAO

The ELISA results are shown in Fig. (**2**). Compared with those in the sham group, the levels of 5-HT in the hypothalamus of rats in the model group were significantly reduced (*p <*0.05). Compared with those in the model group, the content of 5-HT in the EA group increased significantly (*p <*0.05). This result indicated that EA could increase the levels of 5-HT in the hypothalamus of pMCAO rats.

### 3.3 EA can Alleviate the Decrease of 5-HTT Expression in the Hypothalamus Induced by pMCAO

As shown in Figs. (**3A** and **B**), a Western blot was used to detect the expression of 5-HTT in the hypothalamus. Compared with that in the sham group, the expression of 5-HTT in the model group decreased significantly (*p <*0.05). The levels in the EA group were significantly higher than that in the model group (*p <*0.05). This result indicated that EA could alleviate the decrease in 5-HTT expression in the hypothalamus caused by pMCAO.

### 3.4 EA can Reverse the pMCAO-induced Increase in 5-HT2A Expression in the Hypothalamus Induced by pMCAO

The protein expression of 5-HT2A in the hypothalamus of rats in each group is shown in Figs. (**4A** and **B**). The expression of 5-HT2A in the hypothalamus in the sham group rats showed the baseline amount. The expression of 5-HT2A in the hypothalamus in the model group was significantly higher than that in the sham group (*p <*0.05). The expression of 5-HT2A in the hypothalamus in the EA group was significantly lower than that in the mode group (*p <*0.05). It showed that EA can reverse the overexpression of 5-HT2A in the hypothalamus of pMCAO rats.

## 4 DISCUSSION

The hypothalamus, which is a higher subcortical nerve center, is both a neuroendocrine regulatory center and a higher regulatory center of visceral activity. Hypothalamic injury causes pathological changes in some neurons, such as central chromatolysis, acute necrosis of neurons formed by neuronal nuclei consolidation and cytosolic shrinkage and deformation, and possibly the disintegration of myelin sheaths and axons and swelling of astrocytes [2]. These pathological changes can seriously affect neurological function. In addition to these pathological changes in the hypothalamus caused by pMCAO, there are also abnormal changes in neurotransmitters and receptors and transporters, such as 5-HT, 5-HT2A and 5-HTT in the hypothalamus [6]. All these changes can have a serious impact on the nervous system of the organism, leading to neuroendocrine dysfunction and potentially causing multiorgan dysfunction. Therefore, we suggest that the hypothalamic damage caused after pMCAO should be taken seriously. That is exactly why the hypothalamus was used as an important site in this experiment to study the consequences of pMCAO and the effects of EA treatment.

The results showed that pMCAO caused neural tissue damage in the hypothalamus. Moreover, 5-HT, 5-HT2A, and 5-HTT levels in the hypothalamus exhibited abnormal changes. After MCAO injury, the degree of fiber projection at the infarct site was reduced, which could lead to a decrease in 5-HT levels in the distant region, changes in the level and function of other neurotransmitters, and lead to metabolic dysfunction [6]. In contrast, after receiving EA treatment, the 5-HT and 5-HTT levels in hypothalamic tissue were significantly increased, while the abnormal increase in 5-HT2A was significantly suppressed, and the neurological damage in the hypothalamus was effectively alleviated. Therefore, based on the results of this study, the 5-HT system consisting of the 5-HT/5-HT2A pathway and 5-HTT may be a target system for the treatment of pMCAO secondary to hypothalamic injury. This study showed whether the 5-HT/5-HT2A pathway is involved in the EA-mediated treatment of pMCAO secondary to hypothalamic injury.

The results of this study also showed that EA at the GV20 and ST36 points could significantly improve the secondary hypothalamic injury of pMCAO. The combined use of the two acupuncture points was effective at dredging channels and collaterals, modifying the blood and *qi*, and balancing *yin* and *yang* [11, 12]. GV20 belongs to the governor's vessel. After being stimulated, GV20 returns the body to normal functions and disperses local *yang* [13, 14]. ST36 belongs to the stomach meridian, which is rich in both *qi* and blood. Thus, it is considered an acupuncture point that plays a role in the recovery of paralysis [15, 16]. Again, this is one reason why we chose these acupoints.

Previous studies have shown that various drugs or combinations of acupuncture and medicine can affect 5-HT expression in the hypothalamus [17, 18]. However, no studies have reported that treatment of hypothalamic injury secondary to pMCAO with EA could modulate the 5-HT/5-HT2A signaling pathway. In future studies, we can investigate whether 5-HT under EA treatment can exert neuroprotective effects and improve specific signaling pathways associated with neurological injury via 5-HT or further investigate depression-inducing behaviors and solutions induced by increased 5-HT2A expression after cerebral ischemia.

There are also some limitations in this study. First, after stroke in rats, 5-HT2A expression in the midbrain limbic system appears abnormally high in a depression model of rats, indicating that 5-HT2A may be an important target for the treatment of poststroke depression. However, the present study did not verify whether EA could treat poststroke depression by modulating 5-HT2A expression. Recent studies have shown that the expression of 5-HT is reduced in patients with poststroke depression, indicating that EA may treat poststroke depression by regulating the expression of 5-HT [19]. Second, we only focused on 5-HT, 5-HTT, and 5-HT2A in this study. Recent studies have shown that other signaling pathways may also be involved in the effect of EA on cerebral ischemia [20].

Further research is needed to determine whether and how vascular endothelial growth factor (VEGF), Notch, and Hes1 play roles in cerebral ischemia. In addition, it remains unclear whether the combination of EA with exercise training [21] or extracellular vesicles derived from human induced pluripotent stem cells (iPSCs) [22] is more effective. Third, we focused on detecting the levels of 5-HT in brain tissue but did not examine changes in the expression of 5-HT in peripheral plasma. In future research, a model of post-stroke depression will be established, and the combination of EA and other treatment methods can expand the range of detection indicators and further explore the therapeutic effect and potential mechanism of EA on secondary stroke injury.

## CONCLUSION

In conclusion, our results demonstrated that electroacupuncture has a certain therapeutic effect on hypothalamic injury secondary to permanent cerebral ischemia, and the potential mechanism may be closely related to the upregulation of 5-HT and 5-HTT expression and downregulation of 5-HT2A expression.

## Figures and Tables

**Fig. (2):**
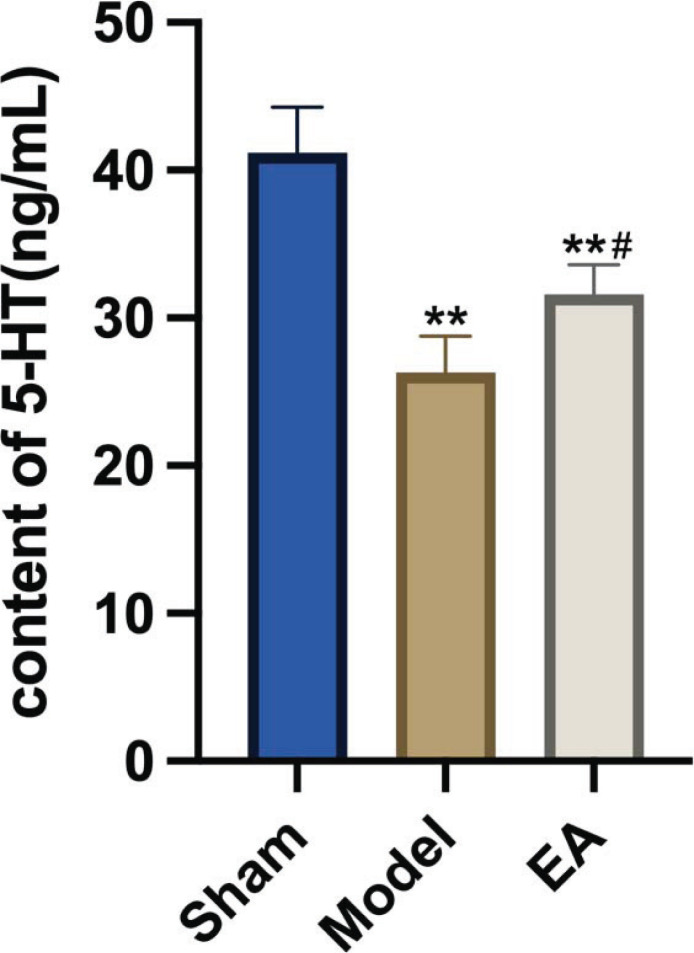
Comparison of 5-HT levels in the hypothalamus of rats in each group (n = 5/group). 5-HT levels in the hypothalamus were determined by ELISA and showed that EA could increase 5-HT in the hypothalamus of pMCAO rats. *vs.* Sham group, **p <0.01; *vs.* Model group, ^#^*p* <0.05. All the results are presented as the mean±SEM. Student’s t-test.

**Fig. (3):**
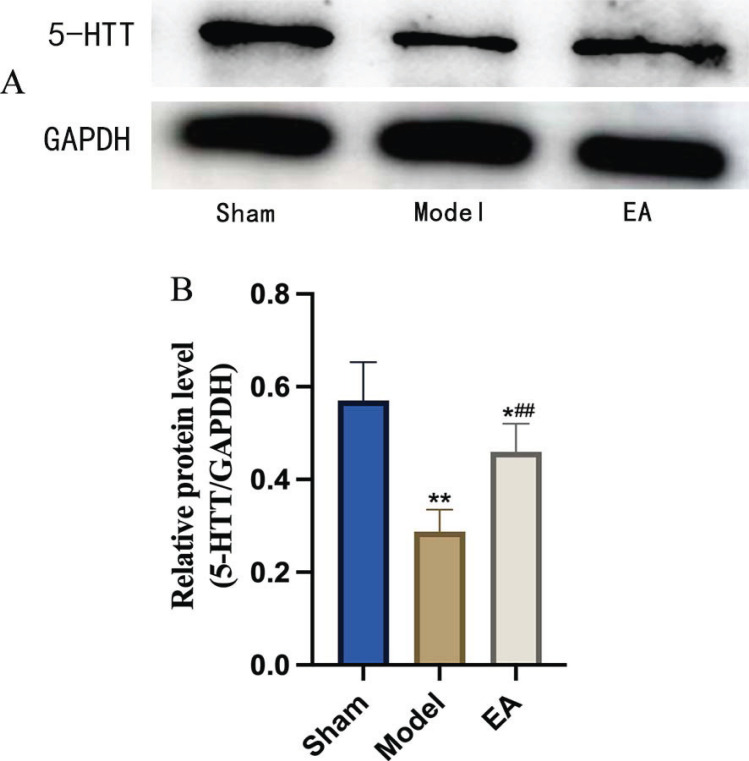
Comparison of 5-HTT expression in the hypothalamus. (**A**) Western blot experimental representative bands of 5-HTT and GAPDH in the hypothalamus of rats in each group. (**B**) Comparison of 5-HTT expression in the hypothalamus of rats in each group (n = 5/group). The results indicated that EA could alleviate the decrease in 5-HTT expression in the hypothalamus caused by pMCAO. *vs*. Sham group, **p <0.01, *p <0.05; *vs*. Model group, ^##^*p* <0.01. All the results are presented as the mean ± SEM. Student’s t-test.

**Fig. (4):**
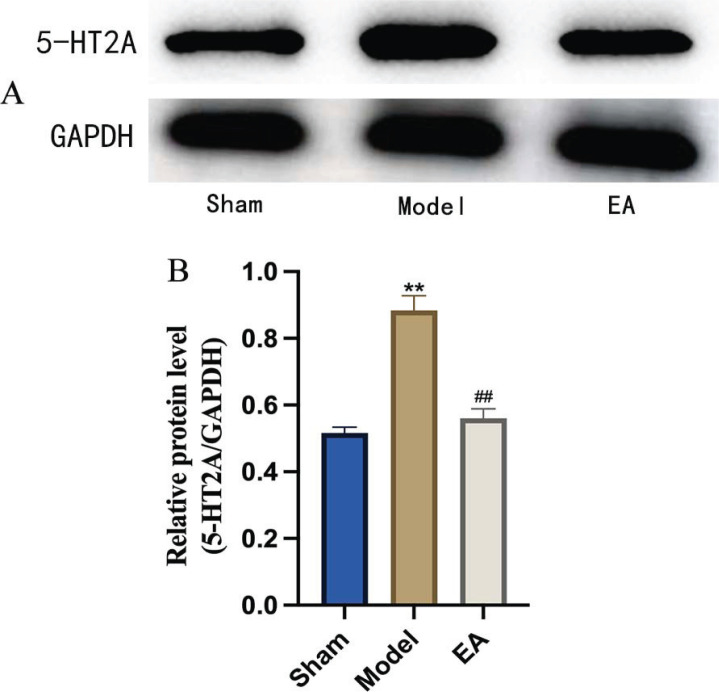
Comparison of 5-HT2A expression in the hypothalamus. (**A**) Western blot analysis of 5-HT2A and GAPDH in the hypothalamus of rats in each group. (**B**) Comparison of 5-HTT expression in the hypothalamus of rats in each group (n = 5/group). The model group showed a significant increase in 5-HTT expression, while the EA group showed a significant decrease. These results indicated that EA could alleviate the pMCAO-induced expression of 5-HTT in the hypothalamus. *vs*. Sham group, **p <0.01; *vs*. Model group, ^##^*p* <0.01. All the results are presented as the mean ± SEM. Student’s t-test.

## Data Availability

The data that support the findings of this study are available from the corresponding author [FW] upon reasonable request.

## LIST OF ABBREVIATIONS

5-HT: 5-hydroxytryptamine
5-HT2A: 5-HT Receptor 2A
5-HTT: 5-HT Transporter
CCA: Common Carotid Artery
EA: Electroacupuncture
ECA: External Carotid Artery
ICA: Internal Carotid Artery
MCA: Middle Cerebral Artery
MCAO: Middle Cerebral Artery Occlusion
pMCAO: Permanent MCAO
tMCAO: Transient MCAO
